# DNA damage activates a complex transcriptional response in murine lymphocytes that includes both physiological and cancer-predisposition programs

**DOI:** 10.1186/1471-2164-14-163

**Published:** 2013-03-12

**Authors:** Cynthia L Innes, Jill E Hesse, Stela S Palii, Beth A Helmink, Abigail J Holub, Barry P Sleckman, Richard S Paules

**Affiliations:** 1 Environmental Stress and Cancer Group, National Institute of Environmental Health Sciences, Research Triangle Park, , NC, 27709, USA; 2 Department of Pathology and Immunology, Washington University School of Medicine, St. Louis, MO, 63110, USA

**Keywords:** miR-155, B cells, Ionizing radiation, DNA damage, Double strand breaks, Transcriptome profiles

## Abstract

**Background:**

Double strand (ds) DNA breaks are a form of DNA damage that can be generated from both genotoxic exposures and physiologic processes, can disrupt cellular functions and can be lethal if not repaired properly. Physiologic dsDNA breaks are generated in a variety of normal cellular functions, including the RAG endonuclease-mediated rearrangement of antigen receptor genes during the normal development of lymphocytes. We previously showed that physiologic breaks initiate lymphocyte development-specific transcriptional programs. Here we compare transcriptional responses to physiological DNA breaks with responses to genotoxic DNA damage induced by ionizing radiation.

**Results:**

We identified a central lymphocyte-specific transcriptional response common to both physiologic and genotoxic breaks, which includes many lymphocyte developmental processes. Genotoxic damage causes robust alterations to pathways associated with B cell activation and increased proliferation, suggesting that genotoxic damage initiates not only the normal B cell maturation processes but also mimics activated B cell response to antigenic agents. Notably, changes including elevated levels of expression of *Kras* and mmu-miR-155 and the repression of *Socs1* were observed following genotoxic damage, reflecting induction of a cancer-prone phenotype.

**Conclusions:**

Comparing these transcriptional responses provides a greater understanding of the mechanisms cells use in the differentiation between types of DNA damage and the potential consequences of different sources of damage. These results suggest genotoxic damage may induce a unique cancer-prone phenotype and processes mimicking activated B cell response to antigenic agents, as well as the normal B cell maturation processes.

## Background

Double strand (ds) DNA breaks are generated in a variety of ways from both genotoxic and physiologic sources. In developing lymphocytes one source of physiological double strand breaks (DSBs) is the process of V(D)J recombination that is utilized to generate rearranged antigen receptor genes [1,2]. This process is initiated by RAG endonucleases while the lymphocytes are in the G1 phase of the cell cycle. These breaks are necessary to create the vast diversity seen in lymphocyte antigen receptors. In addition to physiologic breaks, lymphocytes are exposed to a variety of genotoxic damage from exogenous sources. One such damage source is ionizing radiation (IR), which can be generated from both natural and man-made sources including radon gas and medical devices and procedures. Ionizing radiation can cause DSBs, as well as other DNA lesions, and has been shown to disrupt many cellular functions. Failure to properly repair this damage can lead to detrimental health effects, such as uncontrolled cell death and cancer formation. The normal response to dsDNA breaks includes the activation of multiple transcriptional pathways that can lead to cell cycle arrest at specific checkpoints, DNA repair, or death of the affected cells [3,4]. These genome wide transcriptional responses are very tightly regulated and complex. They also differ between different cell types [5], possibly depending on different sensitivities to DNA damage in general and to different cellular functionalities.

Here we compare the response of developing B cells to both physiologic and genotoxic DSBs. In a previous study we showed that physiological DNA DSBs induced in the G1 phase of the cell cycle by the RAG endonuclease-associated process of V(D)J recombination activated a broad transcriptional profile with many regulated genes involved in diverse processes important for lymphocyte development [6]. While it is known that genotoxic agents, such as IR, activate transcriptional programs involved in maintaining the integrity of the genome, we also want to investigate whether or not the genotoxic breaks could affect lymphocyte-specific maturation transcriptional responses similar to those we observed following RAG-induced physiological DSBs. By comparing the transcriptional responses to both types of DNA damage, we can compare the similarities in the responses to damage as well as the differences induced by genotoxic damage. Similarities in the responses could indicate that genotoxic DNA breaks are potentially disrupting normal cellular functions that occur in developing B cells, thus corrupting these developmental processes. Elucidation of the similarities and differences in these responses may lead to a greater understanding of the cellular mechanisms involved in lympho-proliferative cancer formation and lymphocyte maturation.

In this study we highlight that genotoxic DNA damage not only activates a lymphocyte-specific transcriptional response but also activates a potentially hazardous transcriptional profile that includes a set of genes and pathways indicative of a cancer-predisposition.

## Results

### Ionizing radiation induces a broad transcriptional program in wild type murine pre-B cells

**Figure 1:**
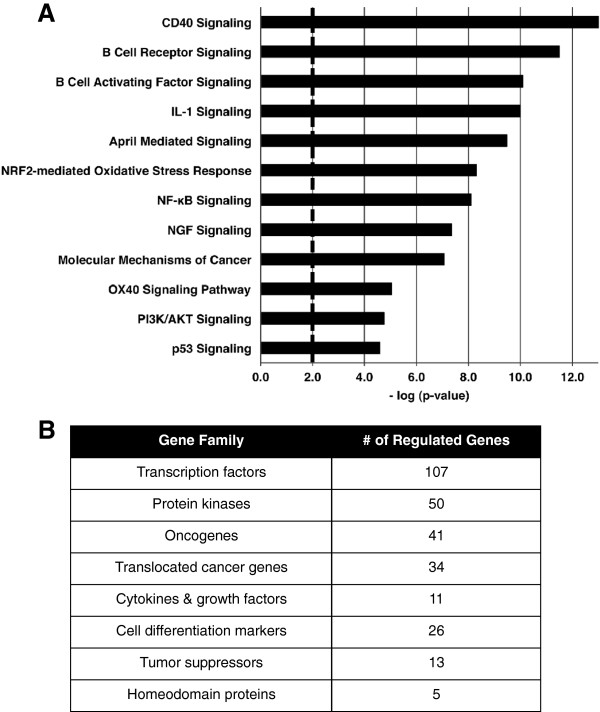
**IR induces robust gene expression changes in G1 phase wild type murine pre-B cells.** (**A**) Some of the significant IPA canonical pathways (pathway significance bar at p-value = 0.01). (**B**) Gene Families from GSEA’s Molecular Signatures Database. These analyses represent the 1940 differentially regulated probes 2 hours following exposure to 1 Gy γ-radiation.

Utilizing Affymetrix whole mouse genome gene expression microarrays, we collected gene expression data from viral (v)-Abl kinase-transformed pre-B cell lines that were generated from multiple WT mice expressing an Eμ-Bcl2 transgene. Treatment of these pre-B cell lines with the Abl kinase inhibitor, STI-571, leads to a block in the G1-to-S cell cycle transition [6]. Cells blocked at the G1-to-S transition with STI-571 were evaluated for their gene expression response to mock treatment or exposure to 1 Gy ionizing radiation (GEO accession GSE36530). The purpose of arresting these cells is to ensure that the cells are in the same phase of the cell cycle as the RAG-induced physiologic response to ensure that similar DNA damage repair processes would be available under both IR and physiologic conditions. We recognize that this will negate detection of some of the gene expression patterns normally identified in logarithmically growing cells that are associated with cell cycle arrest and DNA damage response changes following exposure to IR. In our previous study we provided evidence that the RAG DSB gene expression changes observed in the Abl kinase-transformed cell lines was very similar to that of primary cells. We expect the same to be the case for the IR response. In order to identify significant changes in gene expression after IR-induced DNA damage, we utilized a combination of *t*-test generated p-values and fold change cut-offs (p ≤ 0.05; Fold Change (FC) ≥ ± 1.5) based on global gene expression analysis of 3 WT pre-B cell lines exposed to 0 or 1 Gy IR. We identified 1940 probes that were significantly changed after IR exposure (Additional file 1, column D). Using Ingenuity Pathway Analysis (IPA) [7] and Gene Set Enrichment Analysis (GSEA) [8], we identified a broad range of pathways and gene families with significant responses to induction of genotoxic DNA damage. Figure 1A shows a list of some of the top IPA canonical pathways significantly affected by exposure to IR. The identification of gene families, from the GSEA Molecular Signatures Database, gives a functional overview of the genes identified as significantly altered after IR. Gene families share common features such as biochemical activity and homology. Gene family analysis shows 107 transcription factors as well as 50 protein kinases whose expression levels are significantly altered after IR. This reflects the broad nature of the transcription program initiated by genotoxic DNA damage (Figure 1B).

### Physiologic and genotoxic damage induce a shared lymphocyte-specific response

**Figure 2:**
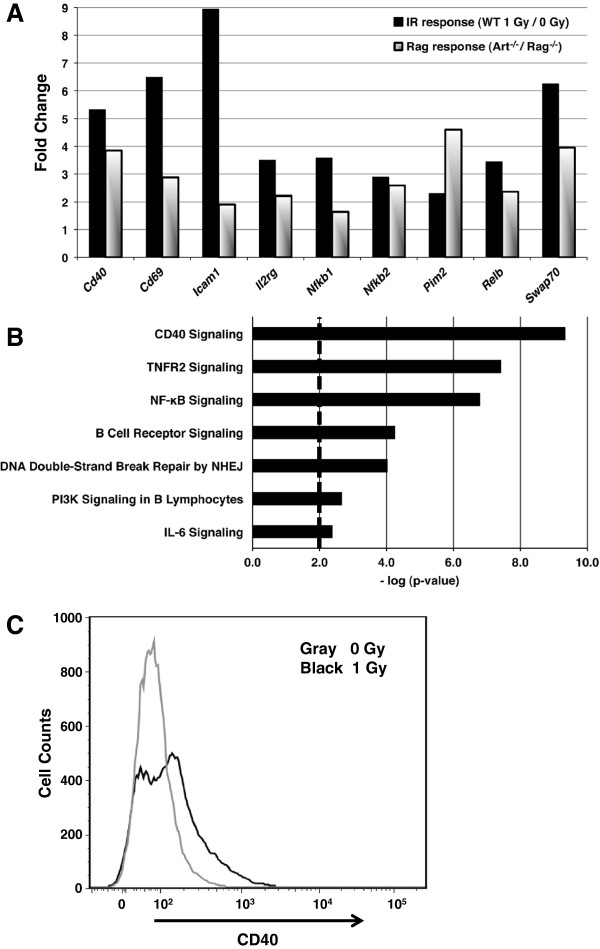
**Both physiologic and genotoxic damage initiate a lymphocyte-specific maturation gene expression response.** (**A**) Fold change from the microarray analysis of representative common genes. (**B**) Some of the significant IPA canonical pathways representing the 288 common probes (pathway significance bar at p-value = 0.01). (**C**) Flow cytometric analysis of CD40 protein expression on STI-571 treated WT cells 90 minutes following exposure to 0 Gy or 1 Gy IR.

In previous work [6], we identified a lymphocyte-specific pattern of gene expression in response to physiologic dsDNA breaks induced by RAG endonuclease cleavage in primary pre-B cells and abl pre-B cell lines. Here we examined the similarities in gene expression changes in response to both physiologic (Additional file 1, column E; GEO accession GSE38044) and genotoxic damage. We identified 288 probes representing genes that are significantly regulated in the same direction in response to both damage types (Additional file 1, column F). Since our WT cells exposed to IR are WT for RAG endonuclease, there may be a contribution of naturally occurring RAG-induced breaks in addition to the IR-induced DNA damage in these cells and the gene expression response may contain elements of responses to both types of DNA lesions. However, we expect the contribution of the RAG-induced breaks to be minor since both the control and irradiated cells should respond in a similar manner to the STI-571 (i.e. not show differential gene expression changes) and since these cells are also WT for the repair pathway machinery that rapidly resolves these breaks. The similarly regulated genes include many lymphocyte specific maturation genes such as *Cd40, Cd69, Swap70*, and *NFκB* (Figure 2A). While these genes are regulated in response to both damage types, one difference we observed was that genotoxic damage appears to induce a more robust change in many of the affected genes and pathways. IPA reveals affected canonical pathways consistent with B cell maturation (Figure 2B). Since CD40 expression plays an important role in B cell maturation and its mRNA levels are increased after DNA damage, we compared the protein expression of CD40 after physiologic and genotoxic damage by flow cytometric analysis. We observed the expected increase in the number of cells with increased CD40 surface expression 90 minutes after 1 Gy IR (Figure 2C) and this is in agreement with our previously published data showing an increase in cell numbers with higher CD40 expression levels after RAG-induced DNA breaks [6].

### Genotoxic damage, but not physiologic damage, induces a potential cancer susceptibility cellular response

**Figure 3:**
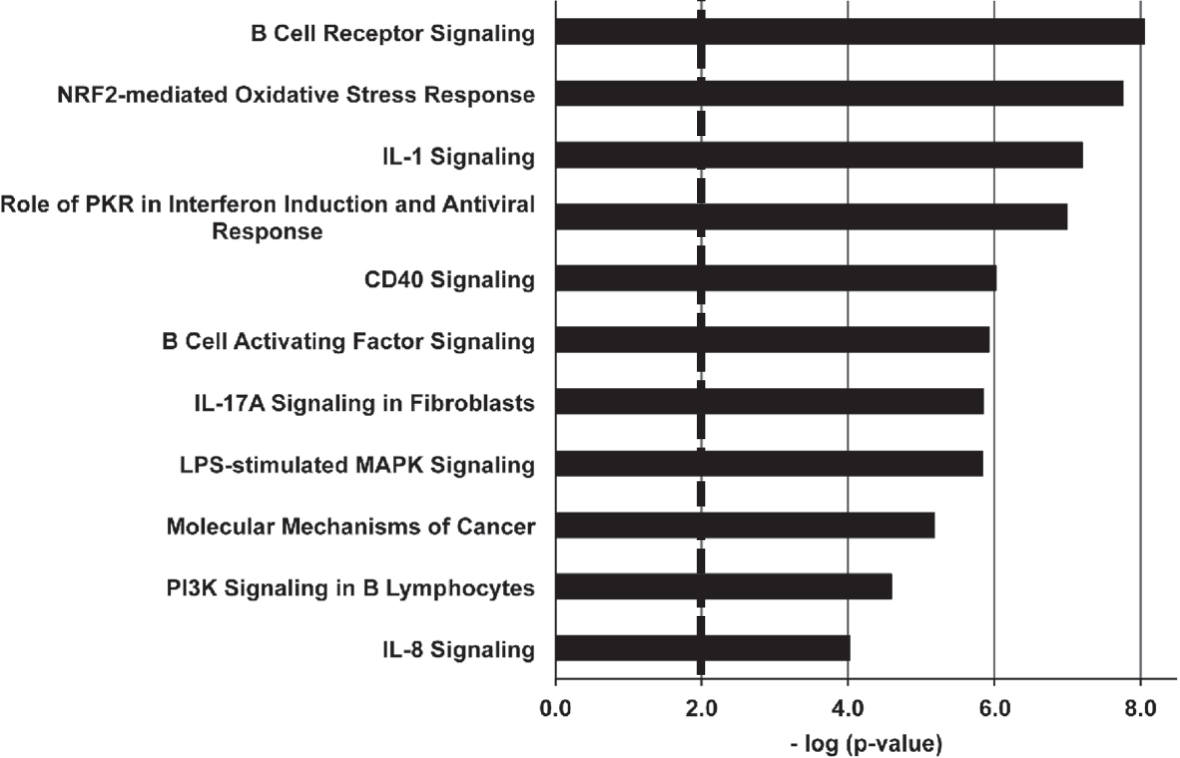
**IPA of 1694 IR-induced only probes.** These are some of the most significant IPA canonical pathways represented by the unique IR-induced genes based on the 1694 probes differentially regulated in response to IR but not RAG-induced damage (pathway significance bar at p-value = 0.01).

**Figure 4:**
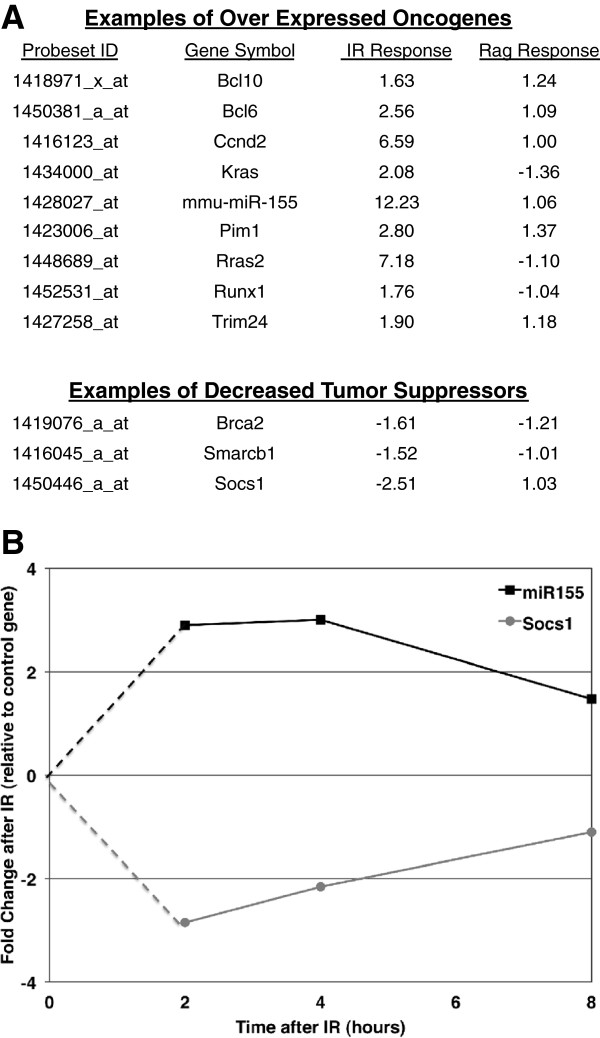
**Increased miR-155 and Kras expression suggest a possible cancer predisposition in G1 phase WT cells.** (**A**) Transcriptional regulation of known oncogenes and tumor suppressors by microarray at 2 hours post IR in WT cells (fold change) (also shown are values of the fold difference after RAG-induced damage). (**B**) Gene expression of miR-155 and *Socs1* mRNA by qRT-PCR at 2, 4 and 8 hours post IR. Each data point is the final ddC_T_ calculated fold change value of 1 Gy/0 Gy from 3 WT pre-B cells lines, with 3 to 5 technical replicates each, following normalization to U6 or 18S, respectively.

In addition to the similarities in response to both types of DNA damage, we observed a robust gene expression profile after genotoxic damage that was not seen after the physiologic damage. We identified 1694 probes, representing almost 900 unique genes, which were differentially regulated in the response to IR but not in the response to physiologically induced damage (Additional file 1, column G). The transcriptional response unique to the IR-induced damage includes increased expression of 24 oncogenes, 25 protein kinases and 57 transcription factors, as well as decreased expression of 5 tumor suppressors and 42 transcription factors. Changes in the expression in these broad-range signalling molecules suggest a diverse biological response to genotoxic DSBs. In order to understand the broad biological mechanism and pathways affected by IR-induced DSBs, we utilized IPA to investigate the changes in pathways and biological functions caused by genotoxic damage (Figure 3). Several canonical pathways were affected in response to IR that were not seen to be affected in physiologic-induced profiles, such as an Nrf2-mediated oxidative stress response and cell cycle regulation pathways. As mentioned above, we also see a stronger enrichment of pathways associated with activation of mature B cells in response to antigen. Initial inspection of the genes significantly regulated by IR-induced damage revealed several oncogenes and tumor suppressor genes whose expression change correlates with changes reported to be involved in cancer formation. These included increased expression of known proto-oncogenes, such as *Kras*[9,10] and *Rras*[10], and the oncomiR microRNA-155 [11-13], as well as suppression of the expression of *Socs1,* a known tumor suppressor [14] (Figure 4A). While the Affymetrix Mouse Genome 2.0 GeneChip array is not specifically designed to recognize microRNAs, the current annotation of the array revealed that several microRNAs are represented in the array. MicroRNA-155 is known to target *Socs1* and this combination of increased expression of miR-155 and suppression of *Socs1* has been described in several B cell-derived lymphomas [11,15]. Our analysis shows many biological functions affected that are consistent with over-expression of potential oncogenes, such as cancer formation, cellular proliferation, and cell-mediated immune responses.

### Increased miR-155 expression suggests an increased capacity for cellular proliferation after ionizing radiation

Increased expression of mmu-miR-155 in response to IR-induced damage suggests an increase in the capacity for proliferation [16] of developing B cells when exposed to genotoxic stress. Mature and functional microRNAs are processed through a series of steps from a primary microRNA transcript. It is possible the change in the mmu-miR-155 levels seen on the microarray is a reflection in the change of the primary miR-155 transcript. In order to investigate the changes of the mature microRNA levels after IR-induced DSBs and to further examine investigate the expression of miR-155 and its target *Socs1*, we undertook a time course to analyze and validate the miR-155 response to genotoxic damage over time. We used quantitative real-time PCR (qRT-PCR) to track the miR-155 and *Socs1* expression levels at 2, 4, and 8 hours post ionizing radiation in the 3 WT lines. A robust increase of miR-155 expression levels is seen in the initial hours after genotoxic damage, with an average of 2.9 and 3.0 fold increases seen at 2 and 4 hours, respectively, post IR. By 8 hours post IR it appears the levels of miR-155 are returning to baseline. In an inverse correlation, *Socs1* expression is robustly decreased initially after IR exposure and has almost returned to baseline levels by 8 hours post damage (Figure 4B, Additional file 2 (miR-155) and Additional file 3 (*Socs1*)).

### Activation of Nrf2 after IR-induced DNA damage suggests a cellular protective response to oxidative stress

One pathway we observed to be regulated after genotoxic but not physiologic damage was the Nrf2 oxidative stress pathway. Members of the Nrf2 signalling pathway, including *Maff*, *Sqstm1*, and *Txnrd1*, were up regulated after exposure to genotoxic DNA damage by microarray. Since IR exposure is known to induce oxidative stress [17,18], induction of this pathway was expected, but in combination with the potential for increased proliferation suggested by miR-155 expression, it may reflect a potentially dangerous, cancer predisposed situation in response to genotoxic damage.

**Figure 5:**
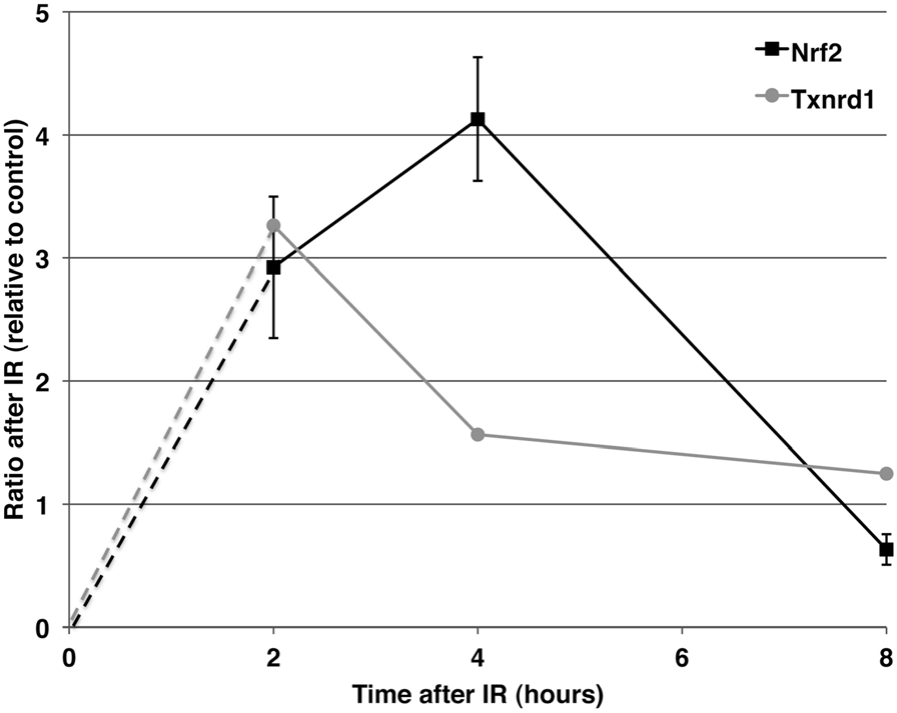
**Activation of the oxidative stress pathway seen in increased Nrf2 and *Txnrd1 *expression following IR.** Nrf2 protein expression was determined by Western analysis and quantification of the average of data from 3 WT cell lines with each Nrf2 intensity pixel value normalized to β-actin. Error bars are standard error of the 3 WT ratios. *Txnrd1* mRNA expression was determined by qRT-PCR and each data point is the final ddC_T_ calculated fold change value from 3 WT cell lines, each with 3 to 5 technical replicates, following normalization to *18*S*.*

In order to investigate and validate the induction of an Nrf2 response to IR, we utilized a time course post IR to examine investigate Nrf2 protein expression levels as well as mRNA expression levels of the downstream target *Txnrd1* in each of the 3 WT pre-B lines (Figure 5 and Additional files 4 and 5). Changes in Nrf2 protein expression were determined by quantitation from Western blots of the 3 WT lines, with each Nrf2 band adjusted for the loading control β-actin. Once activated, Nrf2 protein accumulates in the nucleus, where it transactivates numerous target genes [19,20]. In response to IR exposure, Nrf2 protein levels were found to be on average 3 fold higher than in mock-irradiated controls at 2 hours post treatment. Levels continue to increase, reaching a peak at 4 hours post IR exposure. Levels of Nrf2 are almost completely returned to baseline levels after 8 hours. One downstream target of Nrf2 is thioredoxin reductase 1 (Txnrd1), which plays a role in protecting cells from oxidative stress [21]. As expected, mRNA levels of *Txnrd1*, as measured by qRT-PCR and normalizing to 18S levels, are increased at both 2 and 4 hours post genotoxic damage. By 8 hours the levels of *Txnrd1* are returning to normal levels (C_T_ values are plotted for each WT line in Additional file 4).

## Discussion

In this study we evaluated the transcriptional response induced by physiologic and genotoxic DSBs in developing B cells. By comparing these different types of damage we found that there are important similarities as well as striking differences in the cellular responses to these different forms of DNA lesions.

In previous work [6], we observed a lymphocyte-specific response to physiologically generated RAG-induced dsDNA breaks. Highlights of this response include changes in the expression of genes important in immune function and maturation. Changes in these genes suggest an increase in the signalling of the CD40 and NFκB pathways, suggesting a role for DNA breaks in the progression of B cell maturation. After the observation that RAG-induced DSBs trigger a response to move the B cells toward maturation, the next obvious question is whether or not other types of DSBs induce the same lymphocyte-specific maturation profile. Here we observed 288 probes that were differentially regulated in the same direction after induction of breaks regardless of the source of the damage. These changes include increased expression of *Cd40*, *Cd69*, *Icam1*, *Swap70*, *NFκB*, as well as other immune related genes. Increased expression of these genes and others associated with Cd40-, Cd69-, and NFκB-related pathways suggest that the B cells are preparing to undergo maturation irrespective of the source of the DNA damage. While a core set of genes is regulated in the same direction after both types of damage, the response to genotoxic damage is generally more robust than the response to RAG-induced breaks. We hypothesize this is due to the greater amount of DNA damage induced by IR exposures, perhaps initiating a stronger signal towards maturation.

While we see similarities in the response to physiologic and genotoxic breaks, we recognize there are potential biological and technical differences in comparing RAG-induced and ionization radiation-induced DSBs. RAG-induced DSBs are tightly regulated and only a small number of breaks in very specific locations in the genome are induced. In contrast, IR exposure can induce a broad range of DNA damage, including DSBs, that is not restricted to specific locations but occurs throughout the genome and can involve DNA lesions with damaged nucleotide ends as opposed to the “clean” ends generated by the RAG endonucleases. We have attempted to mitigate these differences by using exposures to a low dosage of IR and by ensuring the cells are in the same phase of the cell cycle to ensure that similar DNA damage repair processes would be available under both conditions. Despite the differences in the nature of the DSBs it appears that the cells undergo a similar response to that damage by initiating a central lymphocyte-specific transcriptional response that is common to both.

In addition to these similarities, the genotoxic damage also induces changes in 1694 unique probes representing almost 900 genes. Broad expression changes in transcription factors and protein kinases suggest genotoxic DSBs induce a myriad of changes in both gene expression and physiological pathways. Gene expression changes and alterations in pathways associated with B cell activation, increased proliferation, and oxidative stress responses are seen in the unique response to IR. Many of the pathways altered on a transcriptional level are known to be involved in the generation of cancers. Also, this study highlights the importance of additional layers of regulation, which has become obvious with the discovery of genotoxic regulation of small regulatory RNAs such as microRNAs (miRNAs). Their role in a wide variety of physiological processes has revealed their vital importance in proper cellular function, and disregulation has been linked to human diseases, including cancer and immune disorders (reviewed in [22-25]).

In addition to the lymphocyte specific transcriptional pattern induced by both types of DNA damage, genotoxic damage induces a potentially oncogenic combination of alterations of genes and biological response pathways. We found that genotoxic, but not physiologic, damage induces increased expression of several proto-oncogenes such as *Kras* and the oncomiR miR-155. This suggested to us that the cellular response to double strand DNA damage could be specific to the method of generation of that damage, recognizing that IR-induced genotoxic damage causes many types of DNA damage. miR-155 is a known oncogenic miRNA and its increase has been correlated with formation of B cell malignancies. The up-regulation of miR-155, at both the primary transcript and mature microRNA level, seen after IR-induced breaks suggests a potential for the development of cancer after genotoxic damage. Additionally, miR-155 has been identified to suppress a number of tumor suppressors, including *Socs1*, which we found to be suppressed after genotoxic damage. MiR-155 up-regulation has been associated with B cell cancers and B cell transformation [11,13], as well as with normal immune response [26]. Altered expression of this miRNA and its target *Socs1* suggests that an increase in proliferation may be triggered after IR exposure. Interestingly, this increase in proliferation and up-regulation of miR-155 has been seen in mature B cells as a result of their response to antigen [27,28].

Another noteworthy difference between genotoxic and physiologic damage is the significant change in regulation of genes in B cell activation pathways and Nrf2-mediated signalling. Nrf2 signalling is a known response to IR but has also been seen in the activation of mature B cells [19,29]. The Nrf2 pathway is a critical regulator of the defense against oxidative stress. Activation of Nrf2 pathways is an important component in the clearing of oxidative stress and in a cytoprotective outcome. There has been some suggestion that Nrf2 also has a role in rescuing cells from cell cycle arrest that can be generated in response to oxidative damage [30]. These responses to genotoxic damage in both the B cell activation and Nrf2 signalling pathways could combine to result in serious deleterious consequences to the immune system.

## Conclusion

The broad gene expression alterations, increased expression of the oncomiR miR-155 and proto-oncogenes such as Kras, the activation of Nrf2, and the lymphocyte specific maturation profile induced by genotoxic DSBs, reflect a potentially dangerous combination of conflicting signals for increased cellular proliferation and cytoprotective responses. These conflicting signals could drive developing B cells to continue to mature and proliferate in the presence of DNA damage after genotoxic exposures. The possibility of maturation and proliferation in the presence of DNA damage increases the risk for aberrant repair or lack of repair of damaged DNA. Continued maturation and proliferation of these highly proliferative cells signalled by genotoxic DSBs provides a mechanism for the development of immunodeficiencies due to the potential loss of mature functional B cells as well as for the formation of lympho-proliferative cancers when DNA repair is not completed successfully.

## Methods

### Cell culture

Three independently derived WT (A70.1, Atm2A, and PA112.2) v-abl-transformed murine pre-B cell lines were used. Cells were maintained in suspension in Dulbecco’s modified Eagle Medium (DMEM), high glucose, (Invitrogen 11960-077) supplemented with 10% fetal bovine serum (Invitrogen 12476-024), 1X Sodium Pyruvate (Invitrogen 11360-070), 1X Non-Essential Amino Acids (Invitrogen 11140-050), 1X L-Glutamine (Invitrogen 25030-081), and 0.0004% β-mercaptoethanol. Treated cells were passaged with 3 μM STI-571 (Imatinib Mesylate) added to the media and incubated for 48 hours. For exposure to ionizing radiation (IR), cells were exposed to γ-rays at a rate of 0.72 Gy/minute for a final dose of 1 Gy from a ^137^Cesium source.

### RNA isolation

Cells were cultured for 48 hours with STI-571 and then for an additional 2, 4, or 8 hours following mock or IR exposure, then were collected and flash frozen. For microarray, RNA was isolated using the Qiagen RNeasy kit following the manufacturer’s protocol, including the addition of DNase. For qRT-PCR analysis, total RNA was isolated using the Qiagen miRNeasy isolation kit using the standard protocol for total RNA isolation.

### Microarray

Isolated total RNA was submitted to the NIEHS Microarray Core facility for microarray analysis. Gene expression analysis was conducted using Affymetrix Mouse Genome 2.0 GeneChip arrays (Mouse 430 v2). One microgram of total RNA was amplified as directed in the Affymetrix One-Cycle cDNA Synthesis protocol. Fifteen micrograms of amplified biotin-complementary-RNAs were fragmented and hybridized to each array for 16 h at 45°C in a rotating hybridization oven using the Affymetrix Eukaryotic Target Hybridization Controls and protocol. Array slides were stained with streptavidin and phycoerythrin using a double-antibody staining procedure, and then washed using the EukGE-WS2v5 protocol with the Affymetrix Fluidics Station FS450 for antibody amplification. Arrays were scanned in an Affymetrix Scanner 3000 and data was obtained using the GeneChip Operating Software (Version 1.2.0.037). The resulting gene expression data from 3 WT pre-B cell lines exposed to 0 and 1 Gy IR were processed and analyzed using Partek Genome Suites (Partek® Genome Suites software, version 6.6beta Copyright © 2009 Partek Inc., St. Louis, MO, USA) utilizing RMA background correction with quantile normalization and eliminating probe sets with an expression level below 100 in all samples. An analysis of variance (ANOVA) was performed between the 0 and 1 Gy treated samples. Associated p-values were generated and, combined with an average fold change of ± 1.5, a p-value of ≤ 0.05 was used to generate a list of differentially expressed genes.

### Flow cytometry

To examine surface protein expression, cells were harvested 90 minutes following irradiation and fixed in 4% paraformaldehyde (BioLegend Fixation Buffer, 420801), diluted with PBS, and stored at 4°C. Cells were stained with CD40-FITC antibody (eBioscience 11-0402) and re-suspended in PBS. Surface expression was determined using an LSRII flow cytometer (Becton Dickinson). Cells were gated for viability based on FSC vs. SSC and the resulting histograms of CD40 (FITC) expression were overlaid for each pair of treated (1 Gy IR) vs. untreated (0 Gy IR) samples using FlowJo (Tree Star, Inc. Ashland, OR) Flow Cytometry analysis software.

### Reverse Transcription and qRT-PCR

#### Quantitative real-time PCR of microRNA-155

Mature microRNAs were measured using the stem loop based TaqMan® MicroRNA Assays kit (Applied Biosystems, Foster City, CA) according to the manufacturer’s protocol. Briefly, microRNAs from 10 ng of total RNA were reverse transcribed with TaqMan® mature microRNA specific stem-loop primers. TaqMan® MicroRNA Reverse Transcription assay kits and reagents were used per the manufacturer’s protocol. Abundance of the microRNAs was measured by qRT-PCR performed on 5^′^-extended cDNA using the Applied Biosystems TaqMan® 2X Universal PCR Master Mix and 5X TaqMan® MicroRNA Assay Mix (mmu-miR-155, MIMAT0000165). For each sample, C_T_ values were obtained from the 3 independent WT cell lines, each with 5 technical replicate wells using an ABI 7900 in the 384 well plate format. MicroRNA concentrations were determined by calculating ddC_T_ with normalization to U6 snRNA. Fold change values were determined based on the normalized ddC_T_ values of the 0 Gy vs. 1 Gy samples. Original C_T_ values of all wells are plotted in Additional file 2.

#### Quantitative real-time PCR for Socs1 and Txnrd1

One-step qRT-PCR was performed using TaqMan Gene Expression Assays (Applied Biosystems) and Superscript II Reverse Transcriptase (Life Technologies). Briefly, 250 ng of total RNA from each sample was combined with Superscript II Reverse Transcriptase, TaqMan gene expression assays (*Socs1*, Mm00782550_s1; *Txnrd1*, Mm00443675_m1) and TaqMan Universal PCR Master mix. 18S RNA was used to normalize gene expression and to calculate the ddC_T_ and fold changes for each gene, as described for miR-155. PCR was run on the ABI 7900 in the 384 well plate format using the following program:

Stage 1 1 cycle 50°C 8 minutes

Stage 2 1 cycle 95°C 10 minutes

Stage 3 40 cycles 95°C 15 seconds

60°C 1 minute

Original C_T_ values of all wells are plotted in Additional files 3 (*Socs1*) and 4 (*Txnrd1*).

### Protein extraction and western blotting

Treated or control cells were harvested by centrifugation, washed 1x with ice-cold PBS, and lysed in IP lysis buffer (Thermo Scientific) supplemented with phosphatase and protease inhibitors (Thermo Scientific). Cell lysates from 3 WT pre-B cells lines were incubated on ice for 30 minutes and cleared by centrifugation at 14 K RPM. Aliquots representing equal amounts of protein (10-30 μg per lane) from each lysate were mixed with sample dilution buffer and denatured by heating at 98°C for 5 minutes, separated on SDS-PAGE gels, and analyzed by western blotting. The antibody to Nrf2 (C-20) (sc-722x) was from Santa Cruz Biologicals (Santa Cruz, CA). Equivalent loading and protein transfer were confirmed by Ponceau stain and Western blot with β-actin (Sigma A5316) as a loading control. Primary antibodies were detected with a peroxidase-conjugated secondary antibody and enhanced chemiluminescence according to the manufacturer’s instructions (Pierce). Quantitation of bands in Western blots was performed with the ImageQuant TL v.2005 software (GE Healthcare).

### Accession numbers

Data from microarrays used in this study have been archived at the Gene Expression Omnibus (GEO) database (http://www.ncbi.nlm.nih.gov/geo/) under GEO accession numbers GSE36530 and GSE38044.

### Additional files

The following additional data files are available with the online version of this paper. Additional file 1 is a table containing data from the microarray analyses. It is a compilation of the probes that were differentially regulated in response to IR-induced breaks in WT cells and in response to RAG-induced breaks. The columns represent the Affymetrix probe ID, gene symbol, gene name, fold change values of probes differentially regulated in response to IR, fold change values in response to RAG breaks, and indicators of which are common to both, or unique to IR-induced breaks. Additional files 2, 3 and 4 contain original **C**_**T**_ values used to determine the changes in transcriptional expression levels from the qRT-PCR of miR-155, *Socs1* and *Txnrd1*. Additional file 5 contains the western blot images for Nrf2.

## Abbreviations

Abl: Abelson tyrosine kinase; Bcl2: B cell leukemia/lymphoma 2; IL1: Interleukin 1 complex; IL6: Interleukin 6; IL8: Interleukin 8; IL17a: Interleukin 17A; NFκB: Nuclear factor of kappa light polypeptide gene enhancer in B cells; NGF: Nerve growth factor; Nrf2: Nfe2l2, nuclear factor, erythroid derived 2, like 2; Ox40: Tnfrsf4, tumor necrosis factor receptor superfamily, member 4; P53: Trp53, transformation related protein 53; PI3K: Phosphatidylinositol 3-kinase; RAG: Recombination activating gene; Tnfr2: Tnfrsf1b, tumor necrosis factor receptor superfamily, member 1b; U6: Small nuclear RNA U6; 18S: Rn18s, 18S ribosomal RNA

## Competing interests

The authors declare that they have no competing interests.

## Authors’ contributions

CLI participated in the conception and design of this project, carried out experiments, analyzed and interpreted the microarray data, and co-drafted the manuscript. JEH carried out the qRT-PCR assays, pathway and functional interpretation of the microarray data, and co-drafted the manuscript. SSP performed the protein assays. BAH and AJH performed primary characterization and growth of the cell lines and provided significant scientific information on growing them. BAH was also involved in early stages of data analysis and interpretation of microarray data. BPS made substantial contributions to the conception and design of the genotoxic portion of this project and participated in revising the manuscript. RSP participated in the conception and design of the project, in the interpretation of the data and in the writing and revising of the manuscript. All authors read and approved the final manuscript.

## Supplementary Material

Additional file 1 **Differentially regulated probes in response to DNA damage.** This is a combined table of all probes that were significantly differentially regulated in response to 1 Gy IR (p ≤ 0.05, FC ≥ ±1.5) in WT pre-B cells lines and in response to RAG induced DSBs (p ≤ 0.05, FC ≥ ±1.5). Three independent WT lines were used to determine the response to genotoxic IR-induced DNA damage. These cells were exposed to STI-571 for 48 hours, treated with 0 or 1 Gy IR and mRNA expression was measured at 2 hours post IR. The intensity values were averaged from the 3 treated or untreated samples and used to determine fold change of 1 Gy/0 Gy. Significantly differentially regulated IR-induced probe fold change values are shown in column D. To determine differentially regulated probes in response to RAG-induced breaks, three independent Rag2^-/-^ (no DSBs) lines and three independent Artemis^-/-^ (unrepaired RAG-induced DSBs) lines were exposed to STI-571 for 48 hours and mRNA expression was measured. The intensity values were averaged for each genotype. Values are fold change of Artemis^-/-^/Rag2^-/-^ and significantly differentially regulated fold change values are shown in column E. Differentially regulated probes common to IR- and RAG-induced break responses are indicated in column F with a # for a commonly expressed specific probe ID and ## for a commonly expressed gene. For the latter, multiple probes representing the same gene were differentially regulated in the same direction (up or down-regulated) following both IR- and RAG-induced breaks. Differentially regulated probes unique to IR-induced DNA damage response are indicated in column G with an *. This column includes all probes that were differentially regulated in the WT response to IR (p ≤ 0.05, FC ≥ ±1.5) but not in the physiological response to RAG-induced breaks. Annotation of all genes listed is based on build 32 from Affymetrix. Click here for file

Additional file 2 **qRT-PCR expression data for miR-155.** Original C_T_ values representing miR-155 and U6 expression levels from 3 WT Pre-B cell lines at 2 (A), 4 (B) and 8 (C) hr following IR are plotted. Data are from 5 technical replicates for each cell line, primer and time point. Click here for file

Additional file 3 **qRT-PCR expression data for *Socs1*.** Original C_T_ values representing *Socs1* and 18S expression levels from 3 WT Pre-B cell lines at 2 (A), 4 (B) and 8 (C) hr following IR are plotted. Data are from 3 to 5 technical replicates for each cell line, primer and time point. Click here for file

Additional file 4 **qRT-PCR expression data for *Txnrd1*.** Original C_T_ values representing *Txnrd1* and 18S expression levels from 3 WT Pre-B cell lines at 2 (A), 4 (B) and 8 (C) hr following IR are plotted. Data are from 3 to 5 technical replicates for each cell line, primer and time point. Click here for file

Additional file 5 **Protein expression of Nrf2 in WT Pre-B cell lines.** Each Nrf2 and β-actin pair of panels comes from the same polyacrylamide gel and represents one of the 3 WT cell lines. The first 3 lanes are untreated cells and the last 3 lanes of each are the corresponding irradiated cells for each time point following IR, 2, 4 and 8 hr. The values in Figure 5 incorporate normalization to β-actin for each lane. Click here for file
