# Supplementary figures and images for: DNA damage activates a complex transcriptional response in murine lymphocytes that includes both physiological and cancer-predisposition programs
Source: BMC Genomics. 2013 Mar 12;14:163. doi: 10.1186/1471-2164-14-163 (PMC3602184; doi:10.1186/1471-2164-14-163)

## Additional File 2

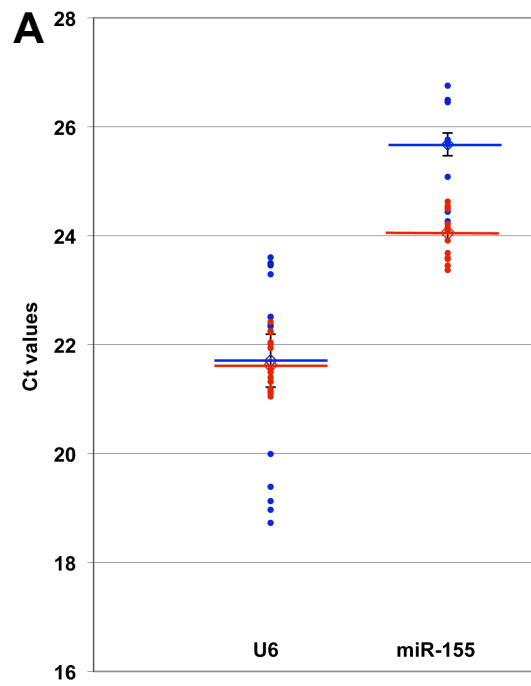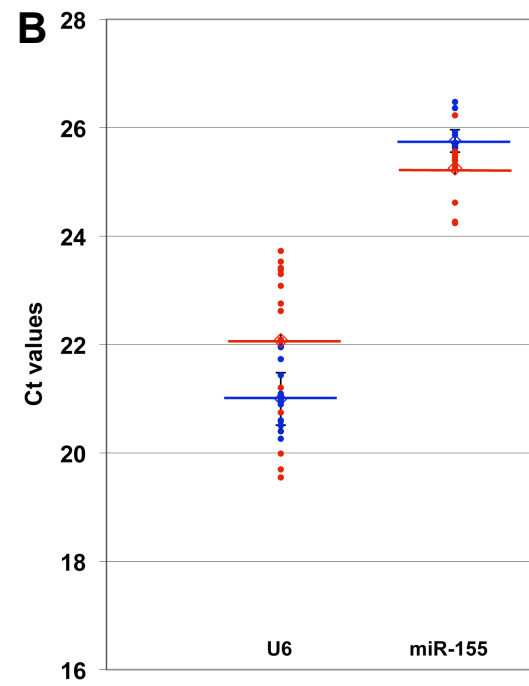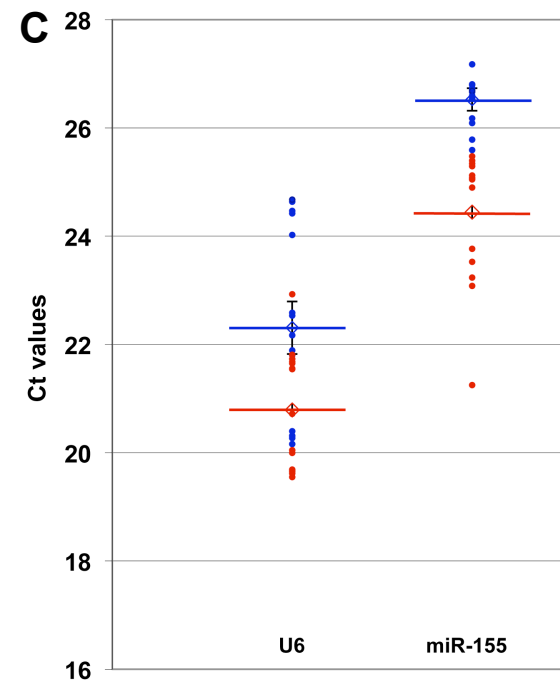

Supplement: Additional file 2 — qRT-PCR expression data for miR-155. Original CT values representing miR-155 and U6 expression levels from 3 WT Pre-B cell lines at 2 (A), 4 (B) and 8 (C) hr following IR are plotted. Data are from 5 technical replicates for each cell line, primer and time point. [file 1471-2164-14-163-S2.pdf]

### Additional File 3

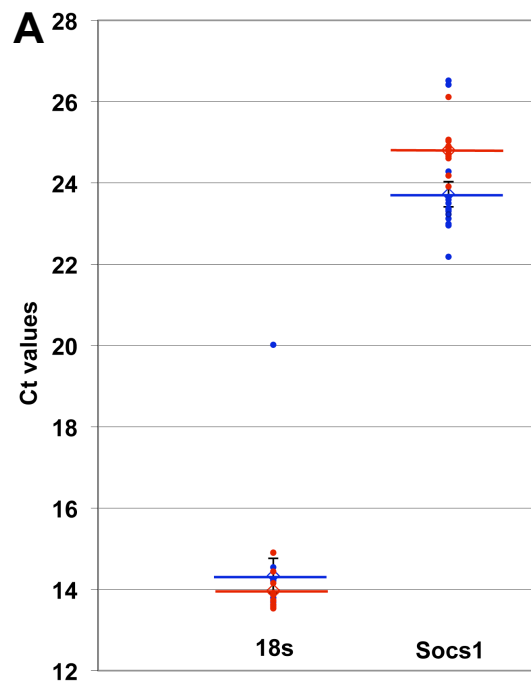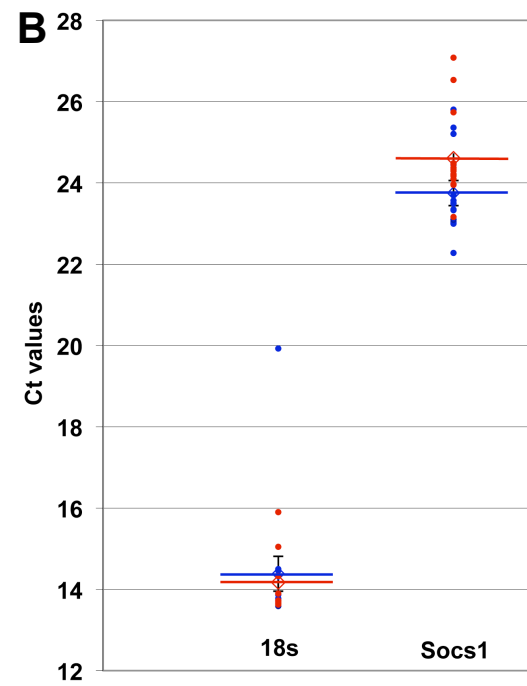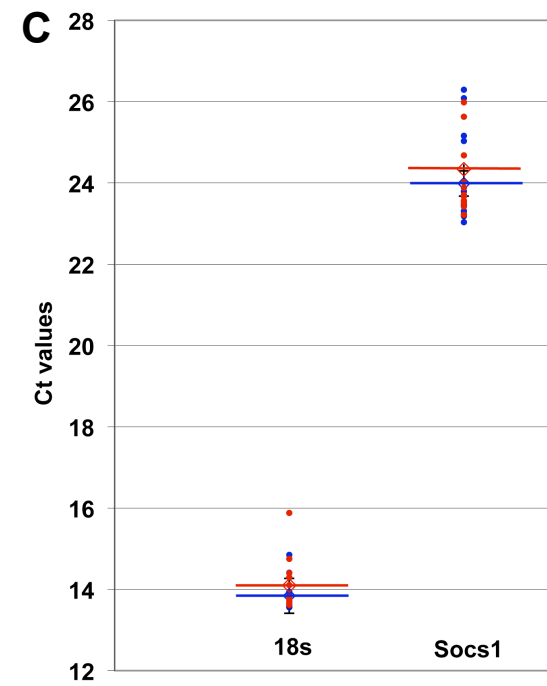

Supplement: Additional file 3 — qRT-PCR expression data for Socs1. Original CT values representing Socs1 and 18S expression levels from 3 WT Pre-B cell lines at 2 (A), 4 (B) and 8 (C) hr following IR are plotted. Data are from 3 to 5 technical replicates for each cell line, primer and time point. [file 1471-2164-14-163-S3.pdf]

## Additional File 4

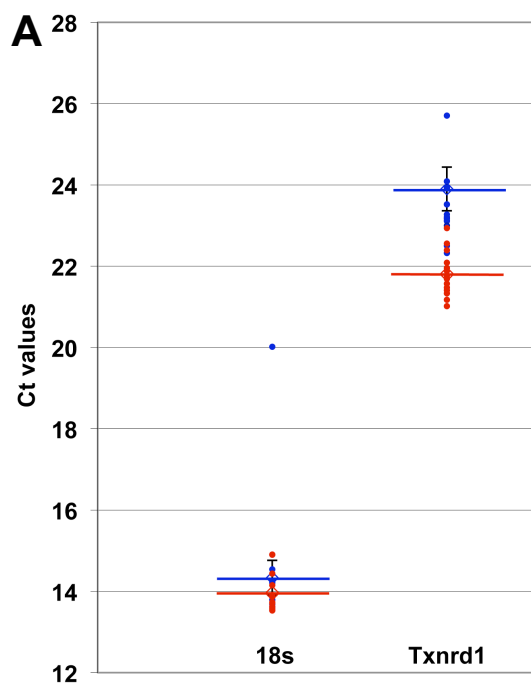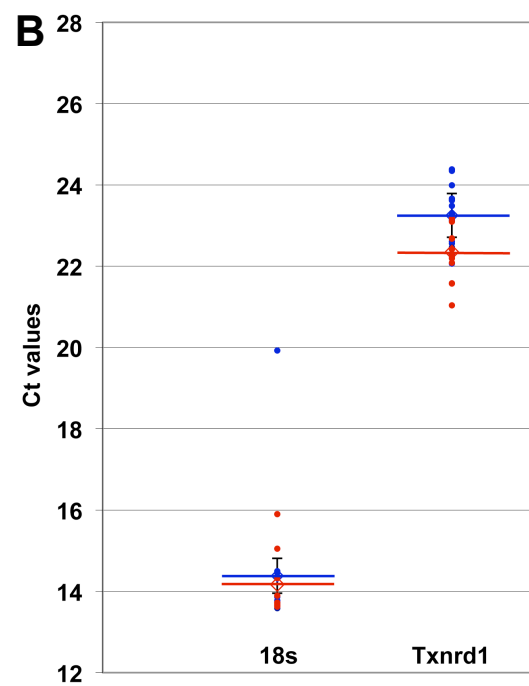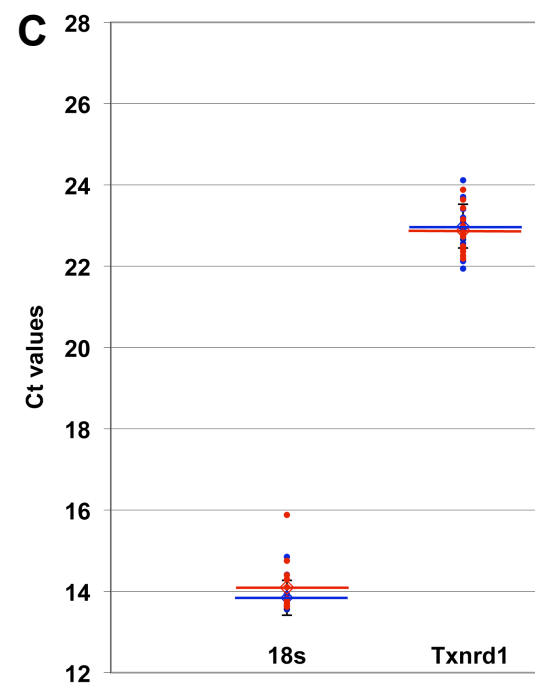

Supplement: Additional file 4 — qRT-PCR expression data for Txnrd1. Original CT values representing Txnrd1 and 18S expression levels from 3 WT Pre-B cell lines at 2 (A), 4 (B) and 8 (C) hr following IR are plotted. Data are from 3 to 5 technical replicates for each cell line, primer and time point. [file 1471-2164-14-163-S4.pdf]

### Additional File 5

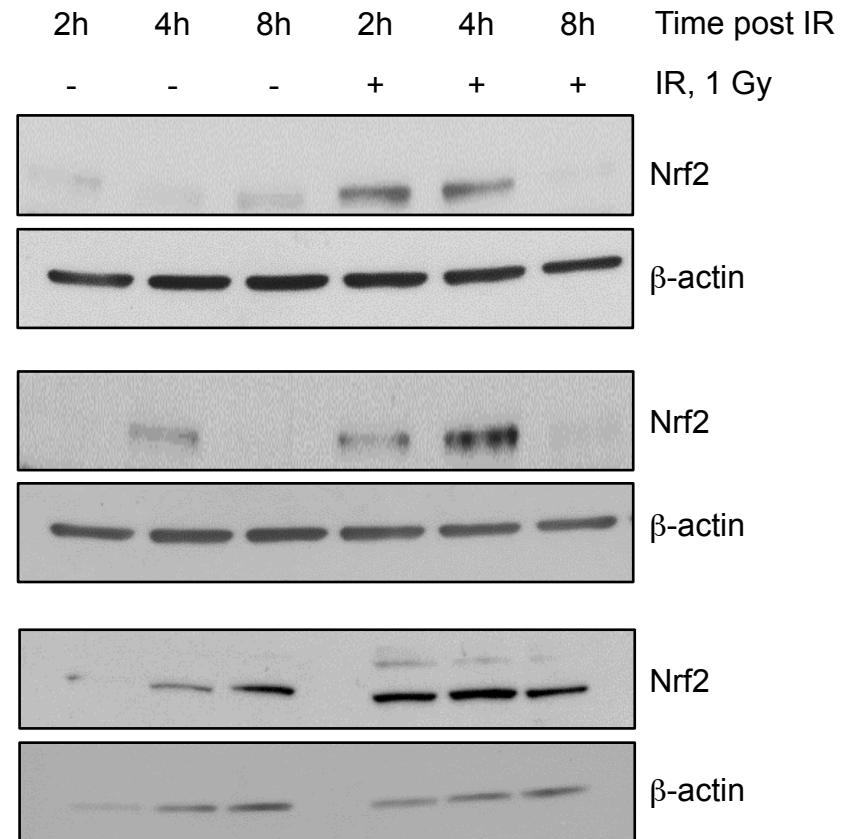

Supplement: Additional file 5 — Protein expression of Nrf2 in WT Pre-B cell lines. Each Nrf2 and β-actin pair of panels comes from the same polyacrylamide gel and represents one of the 3 WT cell lines. The first 3 lanes are untreated cells and the last 3 lanes of each are the corresponding irradiated cells for each time point following IR, 2, 4 and 8 hr. The values in Figure 5 incorporate normalization to β-actin for each lane. [file 1471-2164-14-163-S5.pdf]
